# Role of Metabolic Abnormalities During the Progression of Chronic Kidney Disease and Preventive Strategies

**DOI:** 10.7150/ijms.114382

**Published:** 2025-07-28

**Authors:** Dongqing Zha, Ping Gao, Xiaoyan Wu

**Affiliations:** Division of Nephrology, Zhongnan Hospital of Wuhan University, Wuhan 430070, China.

**Keywords:** metabolic abnormalities, chronic kidney disease, inflammation, oxidative stress, autophagy

## Abstract

Chronic kidney disease (CKD) is characterized by persistent renal impairment or dysfunction that lasts for at least 3 months, and typically has a progressive and irreversible trajectory. The increasing prevalence of metabolic disorders, such as hyperuricemia, dyslipidemia, obesity, and type 2 diabetes mellitus, have contributed to the increasing incidence of CKD, and it is now a significant public health concern worldwide. Accumulating evidence underscores the intricate relationships of the different metabolic disorders and how they promote the initiation and progression of CKD, and ultimately lead to end-stage renal disease (ESRD). Metabolic abnormalities promote CKD progression by various mechanisms, including oxidative stress, chronic inflammation, dysregulation of autophagy, glomerular hyperfiltration and disruption of hemodynamics, endothelial dysfunction, and dysbiosis of gut microbiota. Ectopic lipid deposition and lipid peroxidation-induced redox imbalance lead to mitochondrial dysfunction, excessive production of reactive oxygen species (ROS), and activation of the p38 MAPK, ERK, and JNK signaling pathways. Metabolic dysregulation activates NF-κB signaling pathways and NLRP3 inflammasomes, leading to increased production of pro-inflammatory factors, lysosomal dysfunction, and impaired autophagic clearance, followed by accumulation of metabolic waste and podocyte injury. Obesity and hyperlipidemia can cause excessive activation of the renin-angiotensin-aldosterone system (RAAS), which then causes glomerular hyperfiltration, endothelial and mesangial cell injury and proliferation, and ultimately glomerulosclerosis. Multiple interventions that target these mechanisms have shown therapeutic potential, and these include pharmacological treatments (xanthine oxidase inhibitors to reduce uric acid levels, statins for lipid regulation, and SGLT2 inhibitors and GLP-1 receptor agonists to improve renal and cardiovascular outcomes), lifestyle interventions (low-salt and low-protein diets, weight management, smoking cessation, and alcohol limitation), intermittent fasting, and microbiome-targeted therapies. This review analyzes the pathways by which metabolic abnormalities affect the onset and progression of CKD, identifies strategies that have potential use for prevention or treatment, and offers a robust theoretical foundation for the future development of effective clinical interventions.

## 1. Introduction

Chronic kidney disease (CKD) is defined by impaired renal function that is sustained for 3 months or longer, irrespective of a decreased glomerular filtration rate (GFR), and by distinctive structural or functional abnormalities of the kidneys that are evident on ultrasound. The global prevalence of CKD is greater than 10%, and recent studies projected that CKD will be the fifth leading cause of mortality worldwide by 2040 1-3. Metabolic dysregulation, diabetes mellitus, arterial hypertension, and cellular senescence are the major risk factors for CKD 4-6. However, due to the asymptomatic nature of early-stage CKD, many patients receive delayed diagnosis and treatment, and experience accelerated disease progression. As a result, many patients have advanced-stage CKD at the time of diagnosis, leading to significant healthcare costs and a substantial burden for patients, families, and healthcare systems 7.

The specific types of metabolic dysregulation that lead to CKD include arterial hypertension, hyperuricemia, obesity, insulin resistance, and disruptions of carbohydrate and lipid metabolism (**Table 1**). All of these conditions are affected by the complex interplay among gut microbiota, genetic factors, and nutrition 8, 9. Hypertensive patients frequently exhibit endothelial dysfunction, and chronic endothelial damage can exacerbate glomerulosclerosis and accelerate the decline of renal function and the pathogenesis of CKD 8. Recent researchers have investigated genetic factors related to CKD susceptibility using biobank datasets and clinical repositories, with a focus on the role of hyperuricemia. The findings underscore hyperuricemia as a critical risk factor for the development of CKD 10. A growing body of evidence shows that obesity can drive the progression of CKD and that the pathophysiological mechanisms are multifaceted, including altered hemodynamics, systemic inflammation, oxidative stress, and activation of the renin-angiotensin-aldosterone system (RAAS). Interventions, such as weight reduction and RAAS inhibitors, therefore have important nephroprotective effects 11. Furthermore, studies of rat models of insulin resistance demonstrated that untreated rats experienced significant declines in renal function, along with increased oxidative stress, chronic inflammation, apoptosis, profibrotic remodeling, aggravated renal histopathological damage, and excessive collagen deposition. However, treatment of insulin-resistant rats with vildagliptin (an anti-hyperglycemic drug) led to substantial improvements in renal function, thus emphasizing the critical link between insulin resistance and the pathogenesis of CKD 12. Dysregulation of lipid metabolism also induces renal tubular epithelial cell injury, and this can trigger the release of pro-inflammatory cytokines and the onset of CKD 13.

Given the complex and interdependent relationship between metabolic dysregulation and CKD, prompt treatment of metabolic abnormalities is essential for slowing the progression of CKD, improving patient outcomes, and reducing healthcare expenditures. Diseases related to metabolic abnormalities can accelerate the progression of CKD by activating multiple pathways, such as those that function in inflammation, oxidative stress, inhibition of autophagy, and accumulation of metabolic waste.

## 2. Mechanisms by which Metabolic Abnormalities Promote CKD

### 2.1 Oxidative stress

**Figure 1:**
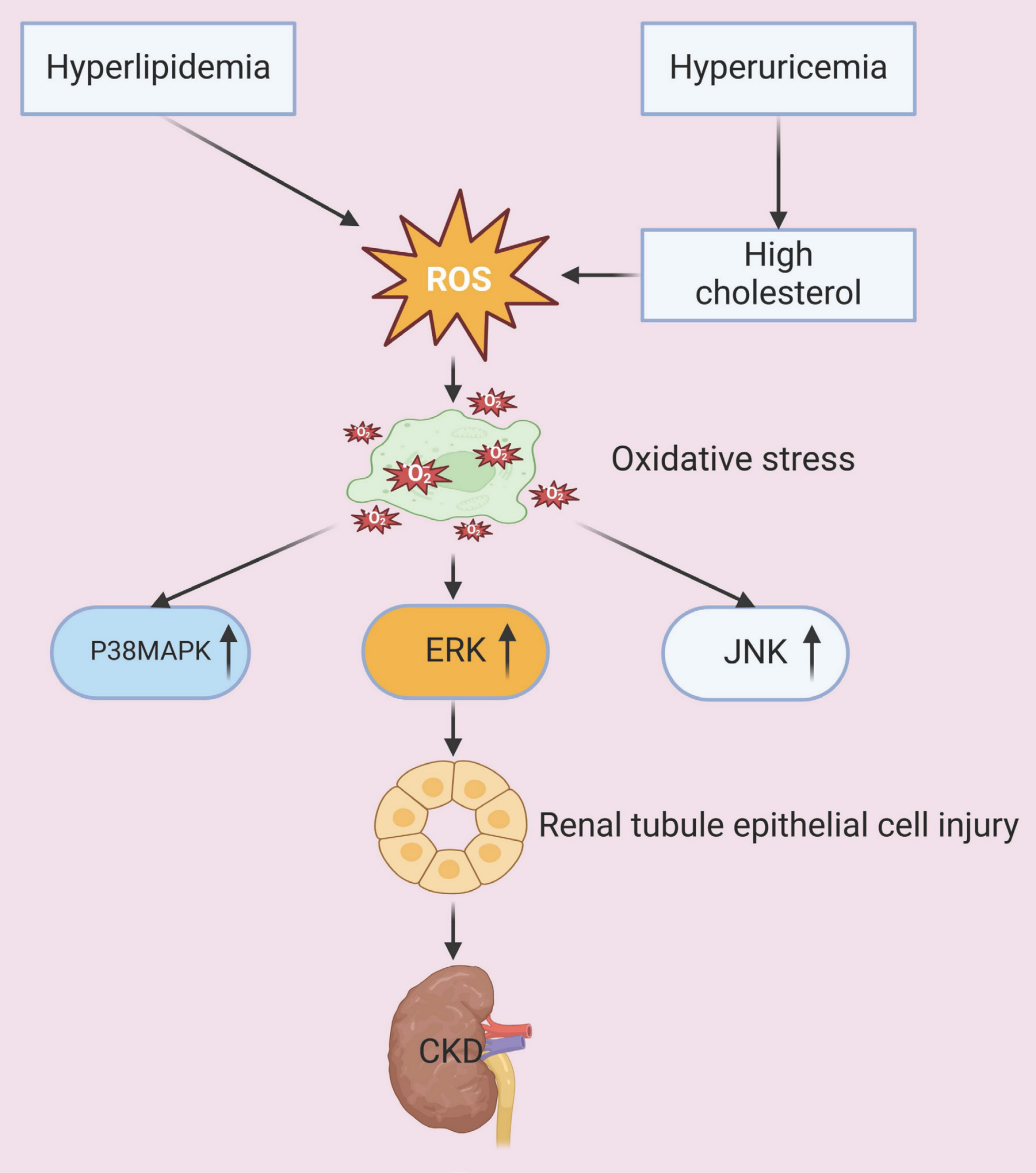
Hyperlipidemia and hyperuricemia increase the production of ROS and oxidative stress. Oxidative stress activates the P38MAPK, ERK, and JNK signaling pathways, which then leads to injury of tubular epithelial cells and accelerated progression of CKD.

The ectopic deposition of excessive lipids in visceral tissues and organs, coupled with a redox imbalance caused by lipid peroxidation, culminates in mitochondrial dysfunction. The resultant overproduction of reactive oxygen species (ROS) induces oxidative damage, which then activates different apoptosis pathways. The peroxidized lipids also disrupt cell membrane integrity and function, and the subsequent oxidative stress causes further damage of membrane lipids, nucleic acids, and proteins, culminating in impaired cellular and organ-level homeostasis 14, 15. Research has also demonstrated that oxidative stress increases the apoptosis of renal tubular epithelial cells by activation of different intracellular signaling cascades, including the p38 mitogen-activated protein kinase (p38 MAPK), extracellular regulated protein kinase (ERK), and c-Jun N-terminal kinase (JNK) pathways. Oxidative stress also exacerbates glomerular hypertension, resulting in dysfunctional endothelial and mesangial cells, disruption of the glomerular filtration barrier, and accelerated progression of CKD 16, 17. An *in vitro* study of human endothelial cells demonstrated that the cytotoxic effects of uric acid can be attributed to an increased production of ROS and angiotensin II 18. The occurrence of this pathological process *in vivo* is associated with increased blood pressure, which promotes the onset and progression of CKD; the co-occurrence of hyperuricemia can also lead to the deposition of monosodium urate crystals within renal tissues, which triggers inflammatory responses, exacerbates oxidative stress, and promotes glomerulosclerosis and fibrosis 19. Additional evidence showed that an elevated level of cholesterol and hyperlipidemia lead to lipid accumulation in renal tubular cells, and this increases the generation of ROS, apoptosis, and inflammatory responses. All of these processes contribute to structural and functional damage of the renal tubules and glomeruli, and also promote the progression of CKD 20, 21. Thus, abundant evidence indicates that certain metabolic derangements, especially hyperuricemia and hyperlipidemia, exacerbate oxidative stress and can lead to renal injury and dysfunction (**Figure 1**).

### 2.2 Chronic inflammation

**Figure 2:**
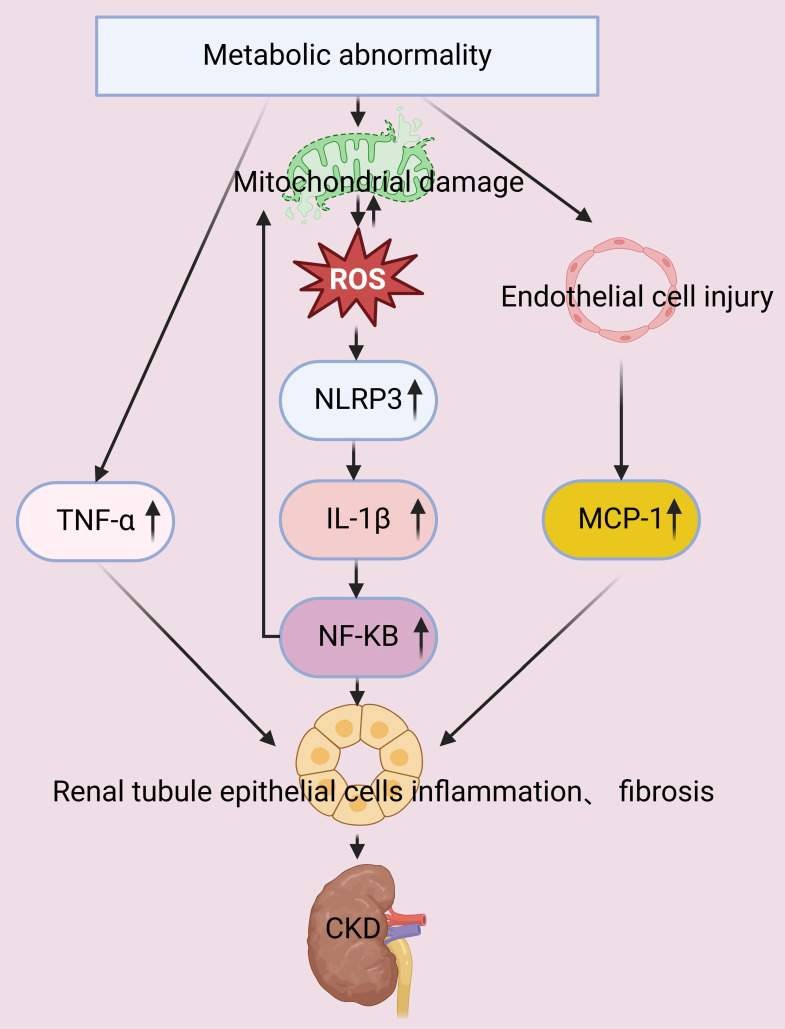
Metabolic abnormalities increase the level of TNF-α and also damage mitochondria and endothelial cells. Mitochondrial dysfunction leads to the production of ROS, activation of NLRP3 inflammasomes, increased expression of IL-1β, and then activation of the NF-κB pathway. These mitochondria-mediated responses, concurrent with the increased levels of TNF-α and MCP-1, lead to injury of tubular epithelial cells and accelerated progression of CKD.

The proinflammatory alterations that occur during metabolic dysregulation contribute to the progression of CKD *via* multiple mechanisms. For example, individuals with obesity or excessive adiposity have an increased risk for upregulation of inflammatory cascades. Clinical investigations have demonstrated that inflammatory mediators, including C-reactive protein (CRP), tumor necrosis factor-alpha (TNF-α), and interleukin-6 (IL-6), play pivotal roles in impairing the renal function of patients with metabolic dysfunction. Renal injury also disrupts the production and secretion of adipokines, thereby promoting the pathogenesis and progression of CKD 22, 23. Proteins in the NF-κB family are pivotal pro-inflammatory transcription factors, and their upregulation leads to mitochondrial dysfunction. The resultant excessive production of ROS activates NLRP3 inflammasomes and other inflammasomes, thereby inducing the production of interleukin-1β (IL-1β). IL-1β then binds to its receptor, and further stimulates the NF-κB signaling pathway, creating a self-amplifying inflammatory feedback loop. This inflammatory cascade also activates leukocytes and resident cells, thus driving the additional production of ROS and reactive nitrogen species. The resulting oxidative stress induces apoptosis, necrosis, and fibrosis, leading to inflamed and dysfunctional kidneys (**Figure 2**) 24, 25.

Thus, chronic inflammation directly impairs renal tissues and contributes to tubular damage, interstitial fibrosis, and impaired renal function. There is clinical evidence that an elevated serum uric acid level has cytotoxic effects on vascular endothelial cells, and the manifestations of endothelial dysfunction include renal vasoconstriction and ischemia-related exacerbations of renal injury. The dysfunction of endothelial cells also activates inflammatory mediators, such as monocyte chemoattractant protein-1 (MCP-1), and this intensifies renal inflammation and promotes renal fibrosis in patients with diabetic nephropathy 26. A study of OLETF (hyperglycemic) rats demonstrated that a high-sugar diet increased renal expression of IL-1β and macrophage infiltration, and that decreasing the serum uric acid level ameliorated albuminuria, tubular injury, macrophage infiltration, and renal secretion of IL-1β. Hyperuricemia in these rats also activated NLRP3 inflammasomes within macrophages, increased chemokine signaling in proximal tubular cells, and promoted diabetic nephropathy due to the interactions of macrophages and proximal tubular cells 27. The chronic inflammatory responses triggered by metabolic dysregulation are therefore critical to the pathogenesis and progression of CKD (**Figure 2**).

### 2.3 Dysregulation of autophagy

Metabolic abnormalities that increase inflammation and oxidative stress also increase the proliferation of glomerular cells, necrosis, glomerulosclerosis, renal interstitial fibrosis, and albuminuria 28. Autophagy is a critical cellular process that facilitates the recycling and removal of damaged proteins, organelles, and pathogens within phagocytic cells. During this process, autophagosomes first encapsulate damaged cell products within their double-membranes; the autophagosomes then fuse with lysosomes, and the resulting phagolysosomes perform the degradation 29. AMP-activated protein kinase (AMPK) is a highly conserved sensor of energy and nutrients in eukaryotic cells that regulates cellular energy and metabolism, and thereby affects diverse biochemical and physiological processes 30. AMPK is also a critical sensor of mitochondrial dynamics, and transmits signals to lysosomes that modulate autophagy. Dual-specificity phosphatase 5 (DUSP5), a protein in the serine-threonine phosphatase family, dephosphorylates ERK and is a pivotal regulator of metabolic signaling, inflammatory responses, and the progression of cancer. Recent studies demonstrated that DUSP5 knockdown reduced the production of inflammatory mediators in renal tubular epithelial cells and also attenuated apoptosis by enhancing AMPK/ULK1-mediated autophagy, leading to improved renal function 31, 32.

These findings demonstrate that an appropriate level of autophagy can prevent renal inflammatory damage, including acute, chronic, metabolic, and age-related insults 33. However, the abnormal accumulation of metabolites that results from metabolic dysregulation leads to lysosome dysfunction in autophagy-associated effector organelles, and this can cause or aggravate diabetes and its complications 34. For example, Yasuda-Yamahara et al. demonstrated that dysregulation of podocyte autophagy contributed to the pathogenesis of diabetic nephropathy. Diabetic patients with severe proteinuria have insufficient podocyte autophagy and significantly decreased podocyte function; similarly, diabetic mice with deficient podocyte autophagy due to a high-fat diet develop extensive proteinuria and marked loss of functional podocytes. The dysregulation of podocyte autophagy therefore plays an important role in the progression of CKD 35.

**Figure 3:**
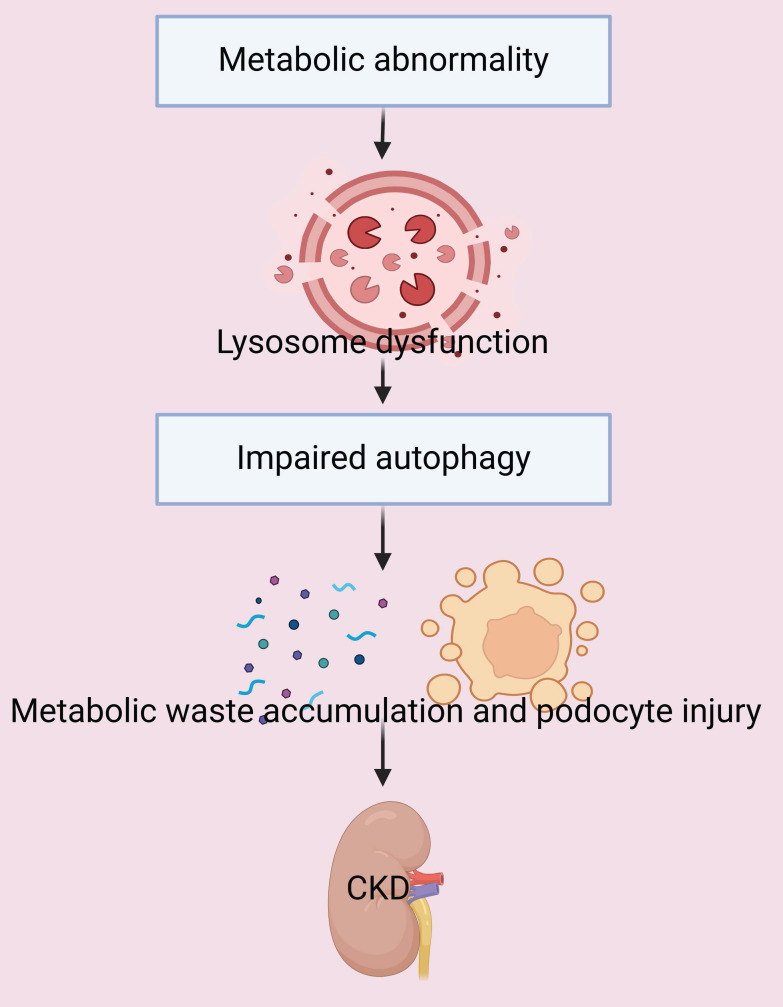
Metabolic abnormalities lead to lysosome dysfunction and impaired autophagy. The disruption of autophagy leads to the accumulation of metabolic waste, causing podocyte injury and accelerated progression of CKD.

There is also evidence that the dysregulation of autophagy due to lysosome dysfunction and impaired autophagic clearance is a hallmark of the accumulation of white adipose tissues in obese mice 36. One of the key mechanisms by which hyperuricemia induces renal tubular injury is by disruption of Na-K-ATPase (NKA) signaling. This disruption triggers inflammation, dysregulation of autophagy, dysfunction of mitochondria, and ultimately renal damage. Notably, activation of autophagy decreases the degradation of NKA in lysosomes, and this decreases inflammation and protects renal tubular cells from hyperuricemia-induced injury 37. In summary, metabolic abnormalities can lead to dysfunctional autophagy and accelerate the progression of CKD (**Figure 3**). This suggests that interventions which restore the normal level of autophagy have potential as therapeutic strategies for treatment of CKD.

### 2.4 Glomerular hyperfiltration and disruption of hemodynamics

**Figure 4:**
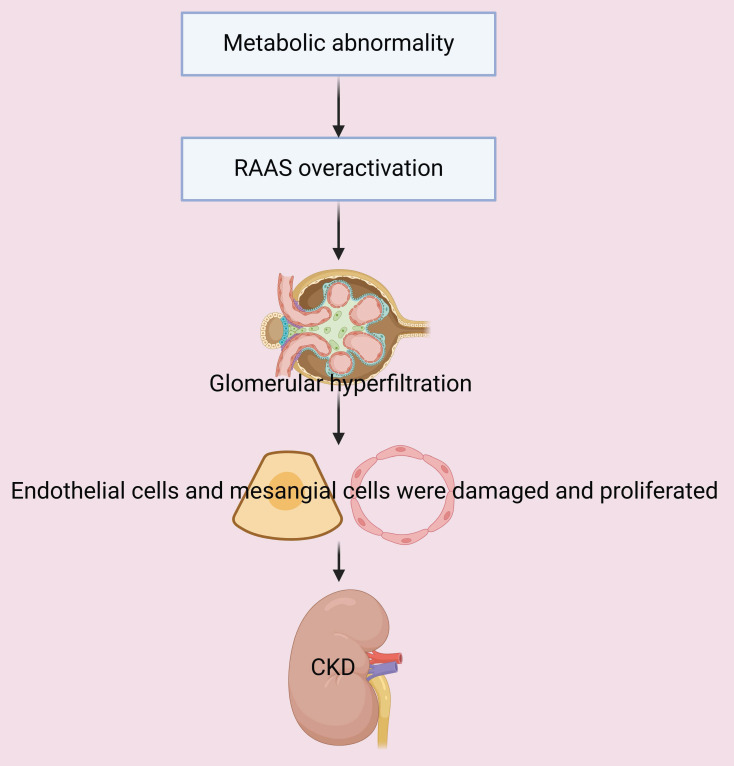
Metabolic abnormalities lead to overactivation of the RAAS. This is followed by glomerular hyperfiltration, damage and proliferation of endothelial and mesangial cells, and accelerated progression of CKD.

Obesity and hyperlipidemia are frequently associated with overactivation of the RAAS, and this leads to increased blood pressure in the glomerular afferent arterioles and renal hyperfiltration. Obesity-induced insulin resistance increases insulin-like growth factor-1 (IGF-1) activity, and also increases glomerular blood flow and hyperfiltration 38, 39. Hyperlipidemia and obesity trigger inflammation, a decreased production of nitric oxide (NO), and an increased level of endothelin, culminating in glomerular vasoconstriction, increased glomerular filtration, and renal dysfunction 40, 41. These responses lead to increased glomerular blood pressure, mechanical stress on the glomerular capillaries, injury and proliferation of endothelial and mesangial cells, and then glomerulosclerosis and the loss of functional nephron units 42. In addition, hyperfiltration leads to excessive excretion of proteins through the glomeruli and proteinuria, and the excess secretion of these proteins has cytotoxic effects, in that it activates inflammatory responses in tubular epithelial cells and induces oxidative stress. This cascade of responses to hyperfiltration increases the production of pro-inflammatory cytokines (TNF-α and IL-6) and free radicals, and decreases the structural and functional integrity of tubular and glomerular cells, thereby accelerating the decline in renal function 43, 44. In summary, metabolic dysregulation injures tubular and glomerular cells and the resulting hyperfiltration and hemodynamic alterations accelerate the progression of CKD (**Figure 4**).

### 2.5 Endothelial dysfunction

Endothelial dysfunction, which is characterized by impaired vasodilation, inflammation, and thrombosis, can trigger cardiovascular diseases and is associated with a decline in the estimated glomerular filtration rate 45, albuminuria, edema, and coagulopathy 46. The intricate relationships among three factors—renal dysfunction, an altered metabolic profile, and adverse cardiovascular outcomes—are a critical area of concern. Mounting evidence underscores endothelial dysfunction and chronic inflammation as driving forces of this detrimental triad. For instance, recent work by Prabhahar et al. 47 and Borri et al. 48 elucidated the specific pathways of endothelial dysfunction in recipients of kidney transplants and aging kidneys, highlighting its profound impact on renal health and systemic vascular integrity. Moreover, the pathophysiology of vascular aging, particularly in the presence of CKD and diabetes, points to shared mechanisms, suggesting that novel cardio-renal protective medications may offer therapeutic benefits by targeting these common pathways 49. A study by Liu et al. 50 also contributed to this understanding by detailing the role of metabolic factors, such as uric acid, in promoting atherosclerosis in patients with CKD, a process intrinsically linked with endothelial damage and inflammation. Collectively, these recent findings reinforce the necessity of considering several interconnected pathologies and underscore the importance of incorporating the latest research to fully appreciate that therapeutic targeting of endothelial dysfunction and inflammation can improve cardiovascular outcomes in patients with renal and metabolic disturbances.

### 2.6 Secondary effects of metabolic abnormalities

**Figure 5:**
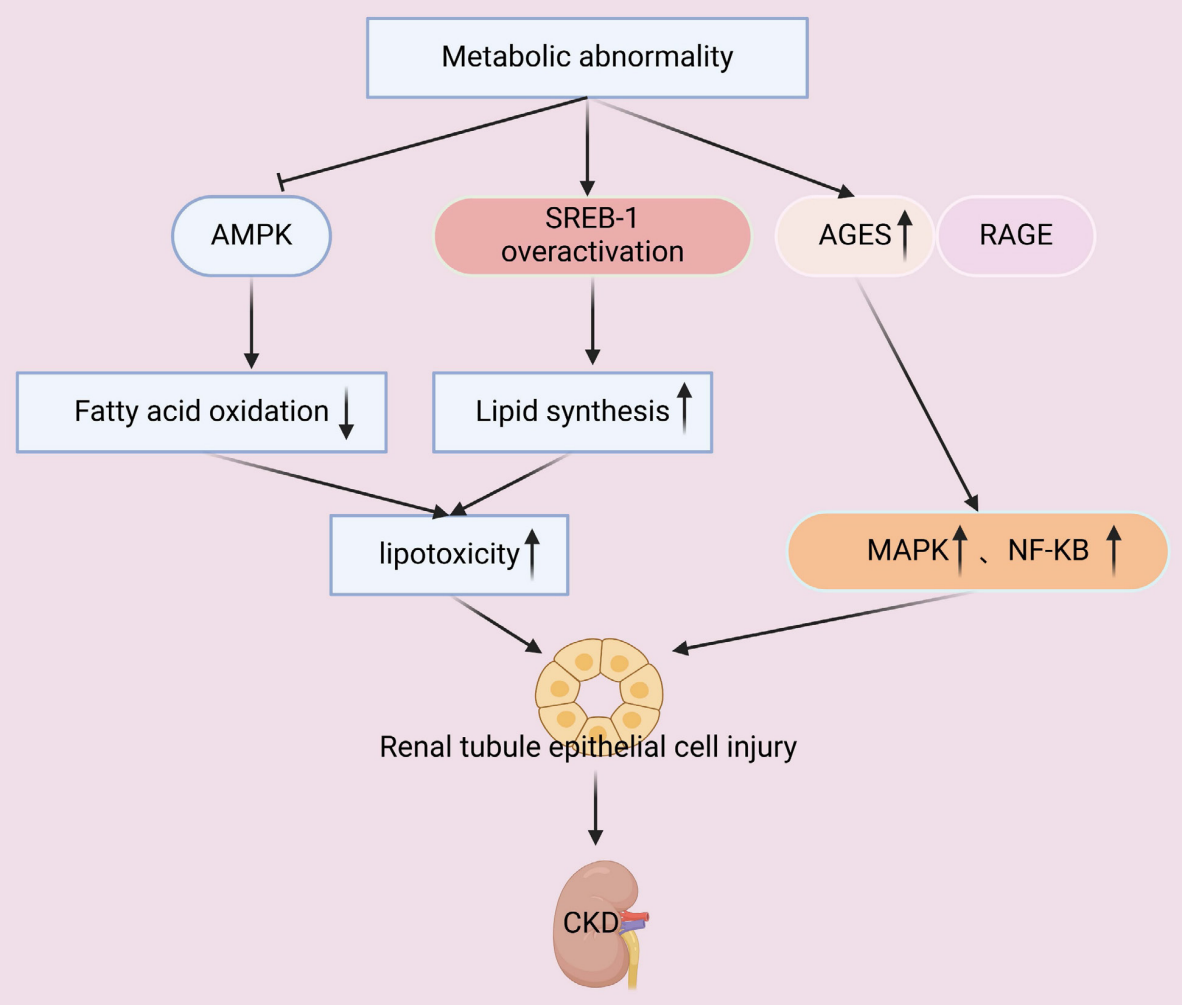
Metabolic abnormalities decrease the level of AMPK, lead to overactivation of SREB-1, and increase the level of AGEs. Suppression of AMPK and overactivation of SREB-1 decreases fatty acid oxidation and increases lipid synthesis, leading to lipotoxicity and injury of tubular epithelial cells. Concurrently, AGEs bind with their receptor (RAGE), and this activates the MAPK and NF-κB pathways. The combined effects of these changes lead to accelerated progression of CKD.

Metabolic abnormalities, such as hyperglycemia and hyperlipidemia, increase the oxidative stress of renal cells and lead to the dysfunction of mitochondria. The resulting excessive generation of ROS has numerous secondary effects, such as damage of mitochondrial membranes, proteins, and DNA (**Figure 2**). Impaired mitochondrial function decreases the production of ATP, so that less energy is available to support the basic functions of renal tubular and glomerular cells, leading to the dysfunction of these cells 51. Metabolic abnormalities can also suppress activation of AMPK signaling in renal cells, and this pathway plays a pivotal role in regulating glycolysis and fatty acid oxidation (**Figure 5**). This dysmetabolism of renal lipids leads to lipotoxicity within renal cells and exacerbates cellular injury 52.

Other studies showed that hyperglycemia leads to the accumulation of advanced glycation end-products (AGEs), and that the binding of these molecules to their receptors (RAGEs) activates the MAPK and NF-κB pathways and increases inflammation and oxidative stress (**Figure 5**). In particular, the cytotoxic effects of AGEs are attributed to their inhibition of glycolysis and mitochondrial oxidative phosphorylation. AGEs also directly damage cellular membranes and disrupt protein structure, and research indicated that this can damage mouse mesangial cells and accelerate the progression of kidney disease in patients with diabetes 53, 54.

Another secondary effect of metabolic abnormalities is that they can stimulate innate renal cells (mesangial and endothelial cells) to upregulate genes that function in lipid synthesis and downregulate genes that function in lipid catabolism, leading to abnormal lipid accumulation and cellular dysfunction 55. In particular, sterol regulatory element-binding protein 1 (SREBP-1) is a critical transcription factor that regulates the synthesis of fatty acids and cholesterol. Metabolic dysregulation leads to over-activation of SREBP-1, and the consequences are increased lipid synthesis and decreased lipid clearance in renal cells, manifesting as lipotoxicity (**Figure 5**). This disrupts cellular membrane integrity and triggers apoptosis and inflammation 52, 56. Disruption of glomerular lipid metabolism also compromises the integrity of the glomerular filtration barrier, leading to proteinuria and impaired renal function. Lipid accumulation within the glomerulus promotes the deposition of extracellular matrix (ECM) and glomerulosclerosis, contributing to the progression of CKD 57, 58. Therefore, further elucidation of the molecular mechanisms responsible for metabolic dysregulation and the abnormal metabolism of renal lipids is essential for developing targeted metabolic therapies for CKD.

### 2.7 Dysbiosis of gut microbiota

Many recent studies have examined the potential impact of gut microbiota on lipid homeostasis of the host and the pathogenesis of CKD. Gut microbiota metabolize a broad spectrum of bioactive lipid metabolites, including short-chain fatty acids (SCFAs) and secondary bile acids, that modulate lipid metabolism and immune responses in the host, and these can affect the progression of CKD 59. Intestinal microbiota ferment dietary fiber and produce SCFAs, such as acetate, propionate, and butyrate. These metabolites are an energy source for intestinal epithelial cells and can also regulate lipid metabolism and inflammatory pathways by activation of G-protein-coupled receptors (GPR41 and GPR43) and inhibition of histone deacetylases 60-63. SCFAs also lower the serum levels of cholesterol and lipids, and this could potentially slow the progression of CKD 64, 65. Furthermore, gut microbiota transforms primary bile acids into secondary bile acids. This suppresses hepatic synthesis of cholesterol and increases cholesterol metabolism and excretion *via* pathways regulated by the farnesoid X receptor (FXR) and the G protein-coupled bile acid receptor 1 (TGR5) 66. Notably, the activation of TGR5 decreases oxidative stress, modulates lipid metabolism, and protects renal function 67. In addition, CKD patients often present with an imbalanced intestinal microflora, i.e., with fewer beneficial bacteria (such as *Bifidobacterium* and *Lactobacillus*) and more harmful bacteria (such as toxin-producing *Clostridium*), and this imbalance can lead to the accumulation of harmful metabolites (such as urinary toxins) that aggravate kidney damage and systemic inflammation 65, 68. Gut dysbiosis can increase intestinal permeability, leading to the translocation of lipopolysaccharides and other bacterial components into the bloodstream, which then trigger a chronic low-grade inflammatory state and contribute to endothelial dysfunction 69-71. Therefore, restoration of the gut microbiota and normalization of lipid metabolism is a potential strategy for slowing the progression of CKD and increasing immune function.

### 2.8 Cross-talk among pathways

**Figure 6:**
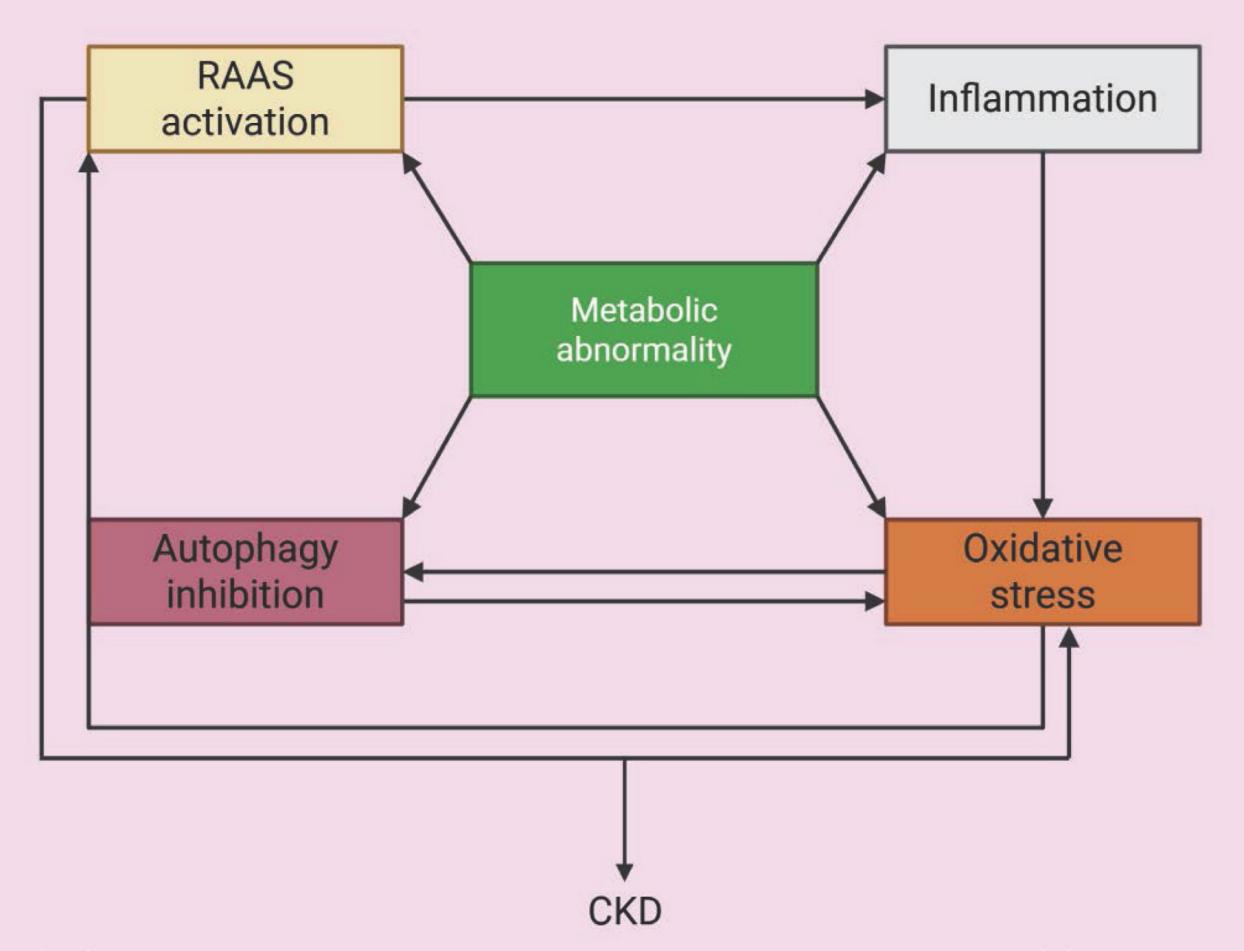
Interactions among pathogenic mechanisms related to metabolic abnormalities. Metabolic abnormalities cause oxidative stress, inflammation, inhibition of autophagy and activation of RAAS, and the interaction of these processes jointly increase the progression of CKD.

The above pathologic processes do not exist in isolation, but are intricately interrelated, forming a complex network of interactions that together drive the development of CKD (**Figure 6**). For example, chronic inflammation is a potent trigger of oxidative stress because inflammatory cells release ROS and the excessive production of ROS activates NLRP3 inflammasomes and other inflammasomes, leading to a self-amplifying inflammatory feedback loop. Both inflammation and oxidative stress severely impair the autophagic process, and the inefficient clearance of damaged organelles and protein aggregates exacerbates cellular stress, inflammation, and renal fibrosis 72. Moreover, stimulation of mitophagy by the mTOR/PINK1/Parkin pathway ameliorates renal inflammation 73. In addition, RAAS dysregulation, which is commonly associated with glomerular hyperfiltration and hemodynamic disturbances, can drive inflammation and oxidative stress within the kidney 43, 44. Furthermore, endothelial dysfunction can be considered a consequence and a trigger of oxidative stress and the inflammatory milieu, and it further compromises vascular health and renal perfusion. Finally, the secondary effects of metabolic abnormalities, such as lipotoxicity and glucotoxicity, can each trigger or exacerbate inflammation, oxidative stress, and autophagy dysfunction. These interactions create a vicious cycle, in which each pathological pathway triggers or exacerbates the others, leading to persistent renal injury, fibrosis, and the progressive decline in renal function, culminating in CKD.

Gender differences also have critical roles in CKD, in that they can affect patient susceptibility, disease progression, and patient prognosis 74. Studies have consistently shown that males develop ESRD more frequently and rapidly than females, and that this difference is partly attributable to differences in sex hormones 75. For example, estrogen can affect cell proliferation, programmed cell death, immune responses, and metabolic regulation 74. An animal study by Ren et al. showed that supplementation with 17β-estradiol (E2), one of the main circulating estrogens in females, protected the kidneys from damage by decreasing inflammation and collagen synthesis 76. Therefore, possible mechanisms responsible for gender differences in the progression of CKD could be differences in the direct effects of sex steroids on the kidneys, differences in nitric oxide metabolism and levels of oxidative stress, and differences in comorbidities and lifestyle-related risk factors 77. Therefore, attention should be paid to gender differences and the above mechanisms.

## 3. Interventions that Target Metabolic Abnormalities

### 3.1 Drugs

A dysregulated metabolism can lead to many adverse effects, and CKD is one of the most consequential because it has a profound impact on physical and psychological well-being. Pharmacological interventions are one of the most common and efficacious approaches for mitigating and managing CKD. For example, clinical investigations have demonstrated that xanthine oxidase inhibitors (allopurinol and febuxostat) lower the serum level of urate, attenuate the progression of renal dysfunction, and reduce the incidence of cardiovascular complications 78-80. Additionally, an animal study showed that febuxostat ameliorated renal interstitial fibrosis by increasing the signaling from bone morphogenetic protein 7 (BMP-7), which inhibits transforming growth factor β (TGF-β) signaling and expression of uterine sensitization-associated gene-1 (USAG-1) 81. Clinical evidence indicates that statins are efficacious and well-tolerated when used to lower the level of serum lipids, and that they can decrease the risk of end-stage CKD and cardiovascular complications following renal transplantation 82. For example, lovastatin slowed the progression of CKD by attenuating oxidative stress, modulating the TGF-β1/Smad signaling, and ameliorating the glomerular endothelial-to-mesenchymal transition in rats with diabetic nephropathy 83.

**Figure 7:**
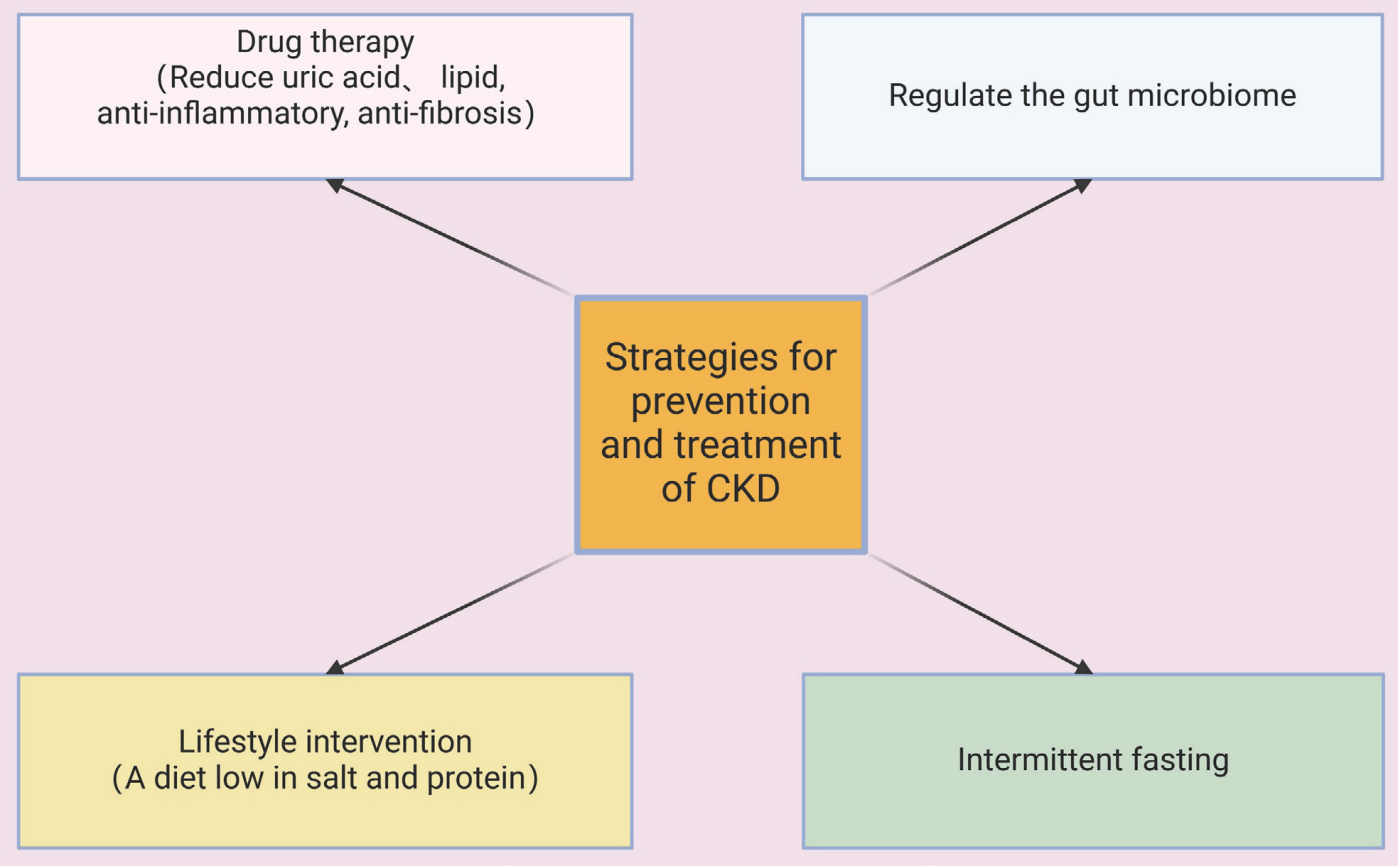
Multiple interventions have the potential to prevent, treat, or slow the progression of CKD. These include medications that lower the levels of uric acid and lipids, and those that have anti-inflammatory and anti-fibrotic effects; lifestyle modifications, such a low-salt and low-protein diet; intermittent fasting; and restoration of the balance of the gut microbiome.

Alirocumab and evolocumab are monoclonal antibodies that target hepatic proprotein convertase subtilisin/kexin type 9 (PCSK9), decrease the circulating levels of atherogenic low-density lipoprotein cholesterol (LDL-C), alleviate endothelial inflammation and atherogenesis, and slow the progression of CKD 84. TGF-β increases fibroblast activation and the accumulation of ECM by activating canonical Smad-dependent signaling and non-canonical pathways, including the mitogen-activated protein kinase (MAPK) and PI3K-Akt cascades, and this accelerates renal fibrosis in CKD 85. TGF-β antagonists (although not yet clinically approved) can slow the progression of CKD by neutralizing TGF-β ligands, inhibiting receptor kinase activity, and disrupting downstream signaling in animal models and patients 86. TGF-β inhibitors also suppress fibroblast transdifferentiation, slow the deposition of ECM, decrease inflammatory responses, enhance tubular function, and reduce proteinuria and glomerulosclerosis 87, 88. Thus, many studies indicated that different therapeutic interventions which decrease the levels of urate and lipids and increase anti-inflammatory and antifibrotic pathways have potential as strategies for the prevention and management of CKD (**Figure 7**).

Metabolic modulation, especially for targeting of uremic toxins, is an important approach that can address metabolic abnormalities and renal dysfunction at the same time (**Table 2**). Sodium-glucose cotransporter 2 (SGLT2) inhibitors and glucagon-like peptide-1 receptor agonists (GLP-1RAs) are two large classes of drugs that can markedly improve renal and cardiovascular outcomes in patients with diabetes and CKD 89. A study of dapagliflozin (an SGLT2 inhibitor) examined its effect on hyperuricemic nephropathy (HN) by examining human biopsy samples, mice with HN, and human proximal tubule cells. The results showed that this drug targeted estrogen-related receptor α (ERRα), thereby activating the ERRα-organic anion transporter 1 (OAT1) axis, promoting the excretion of urate, and inhibiting renal interstitial fibrosis 90. Treatment with tirzepatide, a dual agonist of the GLP-1 receptor and Glucose-dependent insulinotropic polypeptide (GIP) receptor 91, induced significant reduction in body weight in multiple trials, and did not increase the risks of adverse renal events, nephrolithiasis, and acute kidney injury, when compared to a placebo and insulin 92. In addition, tirzepatide is a promising therapeutic option for the treatment of heart failure that provides significant metabolic and cardiovascular benefits 93, including a reduced risk of death from cardiovascular diseases and deterioration of heart failure 94.

Mitochondria-targeted antioxidants, including mitoQ, mitoTEMPO, mitoE, mitoCP, SkQ1, SkQR1, and SS-31, can directly eliminate mitochondrial ROS, alleviate the damage of oxidative stress to DNA, lipids and proteins, and protect the functions of renal tubular epithelial cells and podocytes 95. MitoQ conjugates triphenyl alkyl phosphonium cation with coenzyme-Q, and its administration to mice with diabetes dramatically reversed renal tubular injury, downregulated the oxidative stress in tubular cells, and had a protective effect on podocytes by maintaining mitochondrial fitness 96, 97. The results of a randomized controlled trial showed that a 4-week MitoQ supplement was well tolerated in patients with stage 3-4 CKD, and it also improved large vessel endothelial function, arterial hemodynamics, and microvascular function 98. MitoTEMPO significantly improved renal function and decreased podocyte damage in a rat model of CKD through the inhibition of NLRP3 inflammasomes *via* PINK1/Parkin pathway-mediated mitochondrial autophagy 99. SS-31 promotes oxidative phosphorylation, alleviates mitochondrial oxidative stress, and prevents damage to renal tubules and podocytes 100, 101.

### 3.2 Lifestyle interventions

Although pharmacological treatments can be used to treat or prevent CKD, a multifaceted approach that integrates these treatments with lifestyle modifications can provide even more benefit. Firstly, it is important for susceptible or affected patients to adopt a low-sodium and low-protein diet to alleviate the hemodynamic load on the glomeruli 102. The consumption of potassium- and magnesium-enriched foods should be adjusted according to a patient's residual renal function, and phosphorus intake should be restricted to prevent hyperphosphatemia and secondary hyperparathyroidism 103. Secondly, weight management and structured exercise regimens can also help to slow the progression of CKD. In particular, obesity is a well-established risk factor for CKD, and moderate-intensity aerobic exercise can increase insulin sensitivity, decrease blood pressure and systemic inflammation, and improve cardiorespiratory fitness and overall quality-of-life 104, 105. Thirdly, smoking cessation and avoidance of excessive alcohol consumption are especially important for patients with CKD. Tobacco use exacerbates glomerular sclerosis and microvascular injury, and excessive alcohol consumption increases metabolic stress; discontinuing smoking and limiting alcohol intake significantly lowers the risk of cardiovascular complications 106. In summary, adopting a healthy lifestyle is an effective complementary strategy that can help slow CKD progression and improve overall well-being (**Figure 7**).

### 3.3 Special types of lifestyle interventions

There has been recent interest in the use of intermittent fasting (IF) to modulate diverse metabolic and cellular signaling pathways, including pathways that promote renal dysfunction. IF is associated with certain benefits and risks. For example, it can improve metabolic homeostasis by increasing insulin sensitivity and lipid catabolism, and decreasing chronic inflammation, processes intricately linked to the activation of AMPK and inhibition of the mammalian target of rapamycin (mTOR) 107, 108. Moreover, IF decreases oxidative stress and inflammation in the kidneys by upregulating the nuclear factor erythroid 2-related factor 2 (Nrf2)/Kelch-like ECH-associated protein 1 (Keap1) antioxidant axis and downregulating the nuclear factor-κB (NF-κB) inflammatory pathway. Animal studies also showed that IF modulated the sirtuin 1 (SIRT1) pathway, leading to improved mitochondrial bioenergetics and increased cellular resilience to stress 109, 110.

IF appears to provide nephroprotective effects in CKD due to its induction of autophagy, and this is primarily due to its inhibition of mTOR and promotion of LC3-II (the membrane-associated form of LC3), effects that attenuate glomerulosclerosis and interstitial fibrosis 111. Additionally, IF alleviates inflammatory and oxidative stress by suppressing the formation of AGEs and downregulating RAGE, and this decreases renal tubular injury and interstitial fibrosis 112. Collectively, the multifaceted effects of IF on energy metabolism and autophagy, as well as its anti-inflammatory and antioxidant effects, suggest it has promise as an intervention for management of CKD. However, many studies of IF have focused on animal models, so its effects and precise molecular mechanisms on CKD in humans require further exploration.

### 3.4 Microbiome-targeted therapies

Microbiome-targeted therapies are those that treat diseases by altering the composition and function of the microbiome 113. There are several types of microbiome-targeted therapies that directly affectthe microbiome, such as antibiotics, probiotics, prebiotics, synbiotics, oral absorbents, and fecal microbial transplantation 68, and others that have indirect effects such as enzyme inhibitors, receptor agonists, and antagonists 114. Microbiome-targeted therapies have shown potential therapeutic value in a variety of diseases, including intestinal diseases, metabolic diseases, and kidney disease 115.

Probiotics, prebiotics, and synbiotics modulate the gut microbiome by focusing on increasing glycolytic bacteria rather than proteolytic bacteria 116. Briefly, probiotics are living microorganisms that 117, when consumed in moderation, can provide health benefits to their hosts. Typical strains include *Lactobacillus* and *Bifidobacterium*, both of which support gut health and alleviate dysbiosis in patients with CKD 118. The use of probiotics to treat patients with CKD may be accompanied by cardiovascular benefits, an important consideration because patients with CKD often have concurrent cardiovascular problems 119. Prebiotics are primarily non-digestible components that can have a beneficial effect on the health of the host by selectively stimulating the growth or activity of certain genera of microorganisms in the colon. Fermentation of prebiotics, such as inulin, fructooligosaccharides, resistant starch, and galactooligosaccharides, in the gut produces SCFAs, which can regulate lipid metabolism and inflammatory pathways and potentially slow the progression of CKD 120. Synbiotics are made with various formulations of probiotics and prebiotics that work synergistically to restore intestinal ecology 68. Fecal microbial transplantation involves the transfer of feces from a healthy donor to a recipient 121. To normalize the integrity of the intestinal barrier and reduce the levels of pro-inflammatory metabolites, thereby reducing kidney injury and inflammation 122. However, fecal microbial transplantation entails potential risks, such as transmission of an infection and unforeseen immune responses, and future studies are needed to evaluate its safety.

Future studies should also examine the effect of altering the intestinal microbiota on lipid metabolism as a strategy for the prevention or treatment of CKD 123.

## 4. Clinical Perspectives: Integrating Research into Practice

Nephrologists have a clear road map for implementing targeted therapies that can significantly improve the outcomes of patients with CKD, and this review provided insights into the mechanisms of these different therapies. However, instead of treating specific risk factors separately, a holistic clinical strategy is required due to interactions among different metabolic abnormalities, which include oxidative stress, chronic inflammation, dysregulation of autophagy, and hemodynamic alterations.

### 4.1 Early detection and risk stratification

Nephrologists should prioritize routine screening for metabolic markers including uric acid level, lipid profile, and inflammatory biomarkers (CRP, IL-6, TNF-α) in patients with early-stage CKD or those at risk of CKD. This proactive approach can enable identification of patients most likely to experience rapid disease progression and enable timely interventions to improve therapeutic efficacy.

### 4.2 Personalized therapeutic strategies

Significant evidence supports the use of xanthine oxidase inhibitors (allopurinol, febuxostat) for management of hyperuricemia, SGLT2 inhibitors for control of glucose, and statins for dyslipidemia, and nephrologists therefore have specific pharmacological tools that target the metabolic pathways which drive CKD progression. Rather than treating these conditions separately, an integrated approach that addresses multiple metabolic abnormalities simultaneously is likely to provide the most benefit. The demonstrated benefits of dietary modifications, structured exercise programs, and emerging strategies, such as intermittent fasting, offer nephrologists evidence-based lifestyle interventions to recommend alongside pharmacotherapy. The emerging knowledge of the role of gut microbiota in CKD progression also presents nephrologists with novel therapeutic opportunities. Recommendations for dietary fiber supplementation to promote the beneficial production of SCFAs and administration of probiotics to restore the microbial balance of the gut are two practical applications of this research that can be implemented in clinical practice.

By systematically addressing these interconnected metabolic pathways, nephrologists can move beyond simple management of symptoms toward modifying the course of disease, reducing cardiovascular complications, and improving patient quality-of-life. The key to success lies in early intervention, comprehensive metabolic assessment, and patient education that emphasizes the critical role of lifestyle factors in kidney health.

## 5. Summary and Future Outlook

Metabolic abnormalities can aggravate kidney injury and promote the progression of CKD by altering multiple interrelated pathways that can lead to increased inflammation and oxidative stress, dysregulation of autophagy, glomerular hyperfiltration and disruption of hemodynamics, endothelial dysfunction, and dysbiosis of the gut microbiota. Drug interventions, changes in lifestyle, and altering the microbial composition of the gut can slow the progression of CKD by targeting specific abnormalities in the metabolism of lipids, carbohydrates, proteins, and minerals. Future studies that combine multi-omics technologies (metabolomics, genomics, transcriptomics, and microbiomics) may provide a more thorough understanding of the molecular alterations responsible for CKD and a basis for the development of more effective targeted therapies. Future investigations should also examine the metabolic profiles of patients with different stages and subtypes of CKD, and integrate this information with data about genetic predispositions, lifestyle factors, and metabolomics to formulate more personalized therapeutic strategies. However, because CKD is a chronically progressive disease, studying the long-term impact of metabolic dysregulation on disease trajectory will require substantial resources. Therefore, designing efficient research methodologies and optimizing the allocation of resources remain significant challenges. Although animal studies have identified numerous potential therapeutic targets, translating these findings into clinical applications remains challenging because of the need to consider drug safety and efficacy in humans, as well as cost-effectiveness. We anticipate that future research will surmount these obstacles and achieve significant advancements and identification of interventions that improve the prevention and treatment of CKD, and deliver promising outcomes for patients.

## Figures and Tables

**Table 1:** Impact of diseases that cause metabolic abnormalities in CKD.

Disease	Influence on CKD	References
Hypertension	Damages glomerular endothelial cells and mesangial cells, leading to filtration disorders and accelerating the progression of CKD	Ambroselli et al. (8) Frąk et al. (16) Jia et al. (17)
Hyperuricemia	Induces inflammatory responses, intensifies oxidative stress, and promotes glomerular sclerosis and fibrosis	Gherghina et al. (19)
Obesity	Triggers an inflammatory response and accelerates the progression of CKD	Czaja-Stolc et al. (22) Aamir et al. (23)
Insulin resistance	Leads to oxidative stress, inflammation, and apoptosis, accelerating the progression of CKD	Wahba et al. (12)
Lipid metabolism disorder	Injury of renal tubular epithelial cells accelerates the progression of CKD	Chen et al. (13)

**Table 2:** New therapies for slowing the progression of CKD.

Metabolism-related Therapy	Influence on CKD	Mechanism	Reference
Xanthine oxidase inhibitors	Reduce uric acid level and slow the progression of CKD	Specifically inhibits xanthine oxidase, thereby reducing the production of uric acid	Siu et al. (53) Goicoechea et al. (54) Kohagura et al. (55)
SGLT2/GLP1 dual therapy	Promote the excretion of urate and delay the progression of CKD	Activates the ERRα-OAT1 axis	Alicic et al. (60) Hu et al. (61)
Microbiome transplantation	Regulates lipid metabolism and immune response, and slows the progression of CKD	Activates protein-coupled receptors and inhibits histone deacetylases	Zhang et al. (77) Wang et al. (78) Seljeset et al. (80) Andrade et al. (81)

## Abbreviations

CKD: Chronic kidney disease
ESRD: end-stage renal disease
GFR: glomerular filtration rate
RAAS: renin-angiotensin-aldosterone system
ROS: reactive oxygen species
p38 MAPK: p38 mitogen-activated protein kinase
ERK: extracellular regulated protein kinase
JNK: Jun N-terminal kinase
CRP: C-reactive protein
TNF-α: tumor necrosis factor-alpha
IL-6: interleukin-6
IL-1β: interleukin-1β
MCP-1: monocyte chemoattractant protein-1
AMPK: AMP-activated protein kinase
DUSP5: Dual-specificity phosphatase 5
NKA: Na-K-ATPase
IGF-1: insulin-like growth factor-1
NO: nitric oxide
AGEs: advanced glycation end-products
SREBP-1: Sterol regulatory element-binding protein 1
TGF-β: transforming growth factor β
USAG-1: uterine sensitization-associated gene-1
PCSK9: proprotein convertase subtilisin/kexin type 9
LDL-C: low-density lipoprotein cholesterol
SGLT2: Sodium-glucose cotransporter 2
GLP-1RAs: glucagon-like peptide-1 receptor agonists
GIP: Glucose-dependent insulinotropic polypeptide
HN: hyperuricemic nephropathy
ERRα: estrogen-related receptor α
OAT1: organic anion transporter 1
MAPK: mitogen-activated protein kinase
IF: intermittent fasting
mTOR: mammalian target of rapamycin
NF-κB: nuclear factor-κB
SCFAs: short-chain fatty acids
FXR: farnesoid X receptor
